# Isopropyl Myristate-Modified Polyether-Urethane Coatings as Protective Barriers for Implantable Medical Devices

**DOI:** 10.3390/ma2030719

**Published:** 2009-06-30

**Authors:** Nima Roohpour, Jaroslaw M. Wasikiewicz, Alireza Moshaverinia, Deepen Paul, Ihtesham U. Rehman, Pankaj Vadgama

**Affiliations:** 1 School of Engineering and Materials Science, Interdisciplinary Research Centre in Biomedical Materials, Queen Mary University of London, Mile End Road, London E1 4NS, UK; E-Mail: p.vadgama@qmul.ac.uk (P.V.); 2 Section of Oral Biology, College of Dentistry, The Ohio State University, Columbus, OH 43210, USA

**Keywords:** polyether-urethane, isopropyl myristate, blend, physical properties

## Abstract

Polyurethane films have potential applications in medicine, especially for packaging implantable medical devices. Although polyether-urethanes have superior mechanical properties and are biocompatible, achieving water resistance is still a challenge. Polyether based polyurethanes with two different molecular weights (PTMO1000, PTMO2000) were prepared from 4,4’-diphenylmethane diisocyanate and poly(tetra-methylene oxide). Polymer films were introduced using different concentrations (0.5-10 wt %) of isopropyl myristate lipid (IPM) as a non-toxic modifying agent. The physical and mechanical properties of these polymers were characterised using physical and spectroscopy techniques (FTIR, Raman, DSC, DMA, tensile testing). Water contact angle and water uptake of the membranes as a function of IPM concentration was also determined accordingly. The FTIR and Raman data indicate that IPM is dispersed in polyurethane at ≤ 2wt% and thermal analysis confirmed this miscibility to be dependent on soft segment length. Modified polymers showed increased tensile strength and failure strain as well as reduced water uptake by up to 24% at 1-2 wt% IPM.

## 1. Introduction

Development of a new material for packaging implantable medical devices including micro-systems and electronics is of paramount importance for their extended application in the body. Such materials are required to protect both the body and the implantable devices from adverse effects and to ensure that the device performs acceptably inside the aggressive electrolyte media of the body. As with any implant, biocompatibility, especially of the material in direct contact with the patient’s tissues and body fluids needs to be sustained. In addition regardless of specific application, leachable toxic products from electronic components need to be avoided and these in turn need to be protected from water ingress.

Coatings are essentially used to improve the interface of the implant with functional tissues and other body components [1]. Ideal coatings should have good adhesion to the device with retained mechanical integrity and stability in biological media [2]. Polymeric materials are the most common coatings for microsystems; however ceramics, glasses and metals have also been reported [3]. It was found that soft elastomers such as silicone rubber and polyurethanes were particularly suitable for protecting the irregular shapes of implantable electronics [1,3,4,5,6]. It was reported that silicone rubber has higher water permeability compared with polyurethane which may cause problems for sealing implantable electronics [7]. 

Polyurethanes have been used for biomedical applications for more than 40 years and in particular their biostability has made them an appropriate candidate for chronic implantation [8,9]. Notwithstanding such excellent properties, polyether-urethanes are relatively permeable to gases and water which can cause potential problems. Several methods such as copolymerisation, crosslinking, grafting and blending have been employed to reduce the water permeability of polyurethanes and to enhance their physical properties as well as biocompatibility, but these have only been partially successful [10,11]. 

**Figure 1:**
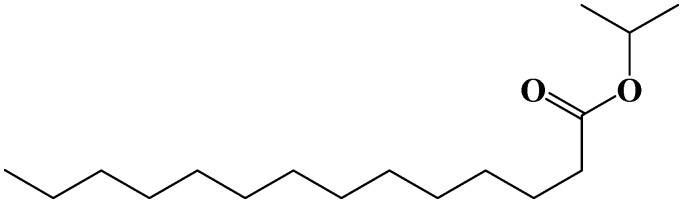
Chemical structure of isopropyl myristate.

In recent decades, amongst all the methods for modification of polymers, blending has received the greatest attention from both the scientific and industrial communities; it is relatively simple, efficient, and offers a low cost route to the establishment of entirely new properties. For biomedical application, the biological response to an additive needs to receive as much attention as the polymer substrate [12]. Non-toxic modifying agents such as lipids have been widely used for surface and bulk modification of biomedical elastomers by respectively grafting [13], blending and end capping of polymer chains [10,14]. For example, phospholipids have enhanced the blood-compatibility of polyurethanes for cardiovascular applications [15,16]. Another type of lipid, notably the synthetic lipid, isopropyl myristate has been used as a plasticizer and a surface active modifying agent in biosensor membranes [17,18]. Isopropyl myristate (IPM) is a long chain hydrophobic ester typically used as the oil component for the preparation of biocompatible micro-emulsions designed to deliver hydrophobic drugs [19,20]. The chemical structure of IPM is shown in Figure 1. 

The packing model presented by Bremner *et al.* [21], showed that blending a certain amount of low molecular weight non-polar polymer with polyether-urethane will reduce its free volume as well as generating a plasticizing effect. We anticipated IPM to have a similar effect on polyurethane. So in this study IPM was introduced in to polyurethane and the properties of the blend assessed over a range of IPM concentrations. IPM at low concentration appears to reduce the permeability of silicone rubber [7], and a similar effect was expected for the polyurethane series. The aim of this work was to establish both better mechanical and water permeability characteristics for polyurethanes and therefore to improve their function as device encapsulants. It was also intended to demonstrate that properties could be enhanced with minimal processing, avoiding new chemistries and associated problems of toxic by products.

## 2. Experimental Section

### 2.1. Materials

Polytetramethylene oxide (PTMO, M_w_~ 1000 and 2000, Sigma-Aldrich, UK) was dehydrated at 80°C in a vacuum oven for 24 hours before use. The hydroxyl value of the polyols was corrected using the method described by Stetzler *et al.* [22]. 1,4-Butanediol ( BD, Sigma-Aldrich ,UK), 4,4’-methylene diphenyl diisocyanate (MDI, Sigma-Aldrich, UK) and dibutyltin dilaurate catalyst (DBTBL, Sigma-Aldrich, UK) were used as received. Tetrahydrofuran (THF, Sigma-Aldrich, UK) and dimethyl-formamide (DMF, Sigma-Aldrich ,UK) were dried over molecular sieves (4Å). 

### 2.2. Synthesis of polyurethane

Polyurethane was synthesised via a two-step, solution polymerization of PTMO, MDI and chain extended with BD. This was carried out in a three-neck flask equipped with a stirrer, a nitrogen inlet and a condenser guarded by a calcium chloride drying tube. The reaction was carried out at molar ratios of (PTMO: MDI: BD) 1:2:1. The temperature of the reaction in the first step was 50 °C whilst all soft segments were dissolved in the DMF. MDI dissolved in DMF was then added drop-wise to the reactor. The temperature was increased to 80 °C and kept for 1 hour to form the prepolymer. The prepolymer was then chain extended using BD at 120 °C for four hours. After cooling to room temperature the copolymer was precipitated in propanol:water (1:1) solution, then washed with methanol and water several times, filtered and dried in a vacuum oven at 80 °C for 24 hour. The hard and soft segment mass ratios in the resulting polyurethanes were respectively 35:65 (PTMO1000) and 25:75 (PTMO 2000). 

### 2.3. Preparation of membranes

Polyurethane and isopropyl myristate (Fluka, UK) were dissolved in THF and stirred for at least 2 hours for the polymer to dissolve. Films were obtained by casting about 25 mL of IPM-polyurethane solution (4 wt%) in THF in glass Petri dishes. Dishes were loosely covered with glass plates, and films dried under forced convection at room temperature for 72 hours. The dried films were then placed in distilled water for an hour before peeling off from the Petri dish and dried in a vacuum oven at 0.1 torr and 80 °C for 24 hours for immediate testing or stored in a dessicator for delayed testing. For analytical studies thinner films were formed by casting on 75 × 25mm glass slides. 

### 2.4. Characterisation

#### 2.4.1. FTIR

FTIR spectra of the synthesised polymers were obtained using a Nicolet 8700 FTIR spectrometer (Thermo Electron Corporation, UK) where the polymer sample films were cast on the KBr crystal to obtain spectra. Spectra were recorded in the mid-infrared region (4000-400 cm^-1^) at 4 cm^-1^ resolution and averaging 128 scans. For presentation, each spectrum was normalized using the area of the 1,412 cm^-1^ peak, assigned to the C-C stretching mode of aromatic ring [23]. Spectral data were acquired using OMNIC 7.2 software. 

#### 2.4.2. Raman

Raman spectra of the samples were recorded using a Nicolet Amelga XR dispersive Raman spectrophotometer (Thermo Fisher Scientific, Madison Wisconsin, USA), equipped with a 785nm laser. All of the spectra were collected in the range 96-3,430 cm^-1^ using a x10 objective and over an average of 128 scans, 1 second exposure time at low resolution. 

#### 2.4.3. Differential Scanning Calorimetry

A Mettler Toledo (UK) DSC823 calorimeter was used in this study. Samples were first heated at 10 °C/min to 110 °C, cooled to -60 °C and then reheated to 220 °C at 10 °C/min.

#### 2.4.4. Dynamic Mechanical Analysis

Dynamic Mechanical Analysis (DMA) was carried out on a TA Instruments Q800 DMA at a dynamic frequency of 1 Hertz, and heating rate of 2 °C/min from -110 °C to 150 °C. 

#### 2.4.5. Mechanical Testing

Tensile strength tests were performed using an Instron Universal Testing machine (model 5584, Instron Co.,UK) equipped with a 10 N load cell at room temperature. Five dog bone shaped specimens per polymer were cut from cast films using an ASTM D638 standard punch. The thickness of the films was 0.1-0.3 mm. The specimens were stretched at a crosshead rate of 20mm/min until rupture. Displacement and load data were gathered from which stress-strain curves were obtained, using the initial cross sectional area of the gauge section and the initial 4 mm gauge length. From this the elastic modulus, ultimate tensile strength (the maximum stress achieved prior to rupture) and percent elongation at break (the strain at rupture) were obtained. 

#### 2.4.6. Water contact angle

Air-water contact angles were measured on polyurethane membranes at 25 °C using the sessile drop method with a KSV CAM200 contact angle instrument (KSV Instruments Ltd, Finland). Drops were formed using a 5 µL Hamilton positive displacement syringe. For this, polymer was cast on a glass slide and dried in a vacuum oven before testing. The contact angle on each side of a drop was measured; the mean contact angles of separate drops are reported here.

#### 2.4.7. Water Uptake

Polymer films were cut to 60 mm × 10 mm dimensions and dried in a vacuum oven for 24h to determine the dry weight (W_d_). Film thickness ranged from 0.3-0.4 mm. Water uptake of the membranes was determined by immersion in deionised water at 37 °C for up to 72 hours. Wet weight at different immersion times (W_t_) was then determined after gently wiping off the surface water with filter paper. Water absorption was then calculated using [24]:

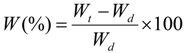
(1)
The mean of five readings was taken.

## 3. Results and Discussion

Polymeric blend materials offer a useful way of extending and adopting existing polymers for biological and other uses. A key practical route is the greater assurance that can be placed on final chemical composition with the lack of reactive by products, as would be the case for modification of the polymer. Several analytical techniques were used to assess the outcome of the blends developed here. The IPM-Polyurethane blends used in this study are listed in Table 1. 

**Table 1:** IPM loaded polyether-urethanes.

Sample ID	Hard segment in PEU (%)	PTMO (Mw)	IPM loading (Wt%)
PEU-1	35	1,000	-
0.5IPM-PEU-1	35	1,000	0.5
1IPM-PEU-1	35	1,000	1
2IPM-PEU-1	35	1,000	2
5IPM-PEU-1	35	1,000	5
10IPM-PEU-1	35	1,000	10
PEU-2	25	2,000	-
0.5IPM-PEU-2	25	2,000	0.5
1IPM-PEU-2	25	2,000	1
2IPM-PEU-2	25	2,000	2
5IPM-PEU-2	25	2,000	5
10IPM-PEU-2	25	2,000	10

### 3.1. Spectroscopy analysis

**Figure 2:**
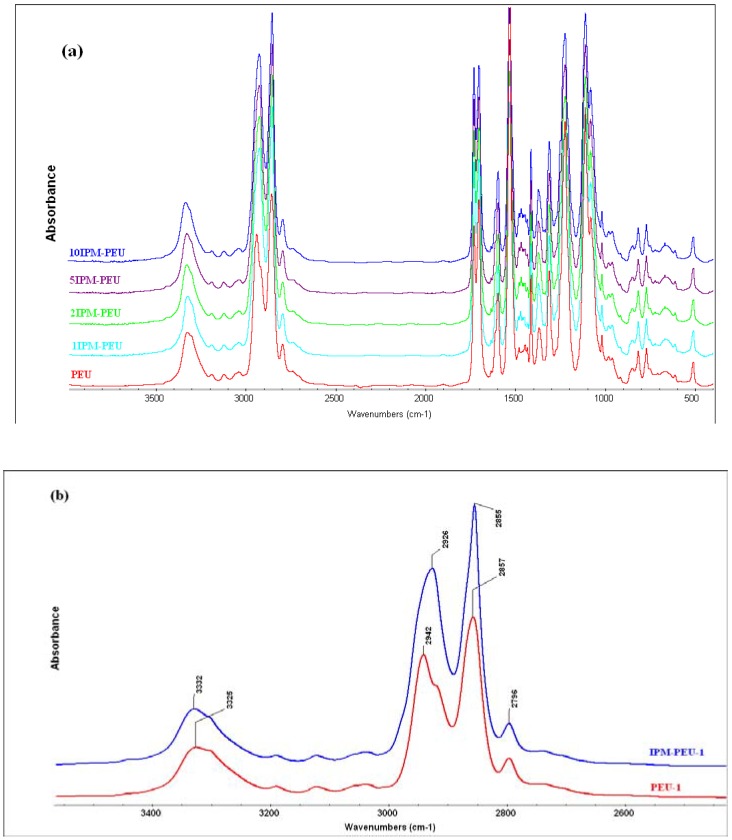
(a) FTIR Spectra of polyether-urethanes loaded with IPM (b) FTIR spectra of IPM-PEU in the range 3,500-2,500 cm^-1^.

**Figure 3:**
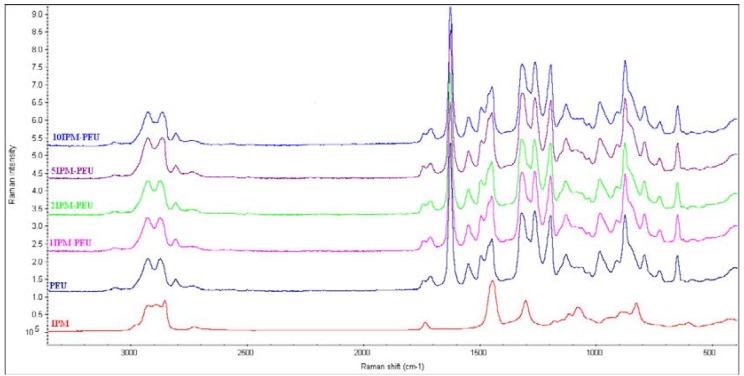
Raman Spectra of polyether-urethanes loaded with IPM compared with Raman spectrum of IPM.

Spectroscopy techniques can be used to study such polymer blends and especially to investigate specific interactions between constituent polymer components [25]. The possible IPM and polyurethane blend interaction was then analysed here by FTIR and Raman spectroscopy (Figure 2 and Figure 3). IR showed a change in the asymmetric bending peak for CH_2_ (2942 and 2857 cm^-1^) by addition of IPM due to the overlap with the asymmetric stretch for CH_3_ in lipid at 2950 and 2920 cm^-1^. Bands at 1465 and 720 cm^-1^ (bending vibrations of CH_2_) and 1250 cm^-1^ (ester) confirmed the registration of IPM in polymer [26]. 

Raman spectroscopy also showed IPM dependant peaks (Figure 3). These are the asymmetric stretch of CH_2_ at 2,930 cm^-1^, the symmetric stretch of CH_3_ at 2,894 cm^-1^, the symmetric stretch of CH_2_ (in lipid) at 2,853 cm^-1^, stretching of ester (C=O) in lipid at 1,734 cm^-1^, CH_2_CH_3_ deformation at 1,448 cm^-1^, CH_2_ deformation in lipid and CH_3_ twisting at 1,299 cm^-1^ [27]. 

Two peaks in the IR spectrum at 1,703 and 1,733 cm^-1^, respectively, represented hydrogen bonded and non-hydrogen bonded carbonyl groups in the PEU. A slight increase in the peak area for the bonded carbonyl is attributed to the formation of hydrogen bonds between C=O group of IPM and the PEU hard segment, and this would suggest a more definitive interaction between IPM and polyurethane. 

 There is also a possibility of hydrogen bonding interaction between C=O and NH of the PEU chains (hard segment) and the polar ester group sites of the IPM. Greater IPM dispersion would facilitate polar and hydrogen bonding interactions. It is well known that FTIR spectra for PEU is sensitive to hard domain organisation and to urethane and urea hydrogen bonding [28,29]. Accordingly, the FTIR spectrum of the IPM loaded polymer was compared with plain polyurethane. Both NH and C=O absorption peaks showed a contribution of overlapping bands related to free and hydrogen bonded groups. PEU had a characteristic major peak at 3,325 cm^-1^ and a small shoulder at 3,195 cm^-1^ due to the absorption of H-bonded N-H, and a shoulder at 3,420 cm^-1^ assigned to free N-H. When the IPM content was increased, the peaks representing hydrogen bonded N-H shifted to higher wave numbers (Figure 2). This shift could be due to hydrogen bonding between the NH group of the PEU and the ester group of the IPM, as the absorption bands of NH group are sensitive to hydrogen bond formation. It is concluded that while hydrogen bonds initially exist within the N-H groups, there is an increase due to new bonding between -NH groups and the ester of the IPM, so leading to a slight shift of the peak representing hydrogen bonded N-H. A similar phenomenon is exhibited in the FTIR spectra in the 1,600-1,800 cm^-1^ region. Two peaks at 1,703 and 1,733 cm^-1^, respectively, represent hydrogen bonded and non-hydrogen bonded carbonyl in PEU. Some inter-molecular hydrogen bonding would help to retain IPM within the PEU, but this structural benefit needs to be confirmed. 

### 3.2. Thermal analysis

**Figure 4:**
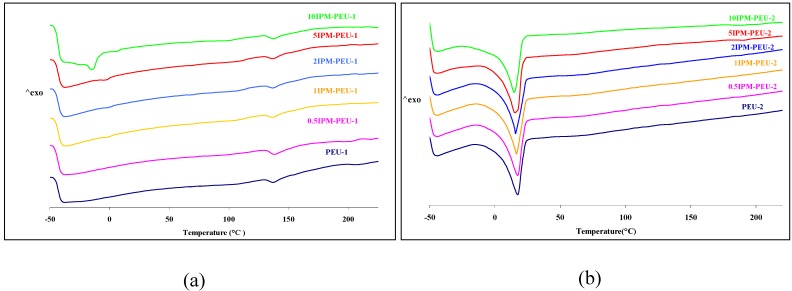
DSC thermogram of (a) IPM-PEU-1 series (b) IPM-PEU-2 series*.*

Figure 4 presents DSC thermograms of both the PEU-1 and PEU-2 series with different levels of IPM. It was found that blends of PEU-1 with more than 2 wt% IPM, had a small endothermic peak at *ca*. 10 °C, due to the melting of IPM, confirmed using DSC scans of pure IPM (data not shown). The segregated peak, moreover, shows that above 2 wt% the IPM exists as a separate phase in PEU-1. The absence of a specific IPM melting endotherm in the PEU-2 series can be explained by its greater miscibility. In PEU-1, with the lower PTMO content, the soft segment becomes saturated with IPM when more than 2 wt% is loaded, and thus a transition to free IPM is observed. 

DSC measurements showed that the melting transition of the hard segment (*ca.* 137 °C) was constant across the range of IPM concentrations. This is consistent with the known stability of crystalline regions in polyurethanes in the presence of plasticizer [30]. 

A sensitive measure of change in packing, and hence free volume, is the glass transition temperature (T_g_). An increase in T_g_ value could be due to a greater packing of polymer chains [31]. With such increased packing, reduced free volume is available for chain motion, so the T_g_ of PTMO would be expected to be shifted towards higher temperatures. Such higher packing would also be expected to reduce the water permeability of the polymer. In this study IPM was expected to act as a plasticizer in the PEU, as it did in PVC [32,33]. The most widely accepted mechanism for plasticization is based on free volume theory [31]. Free volume can be defined as the difference between polymer volume as defined by Vander Waals radii and the total volume occupied by a polymer; this difference is, therefore, the volume associated with the packing of the polymer chains. At or below the T_g_, the polymer will have a limited free volume because large scale molecular motion is partial, and addition of a small plasticiser molecule (with a greater free volume than the polymer, i.e. a lower T_g_), will increase the free volume of the polymer, so decreasing the T_g_ of the blend.

**Figure 5:**
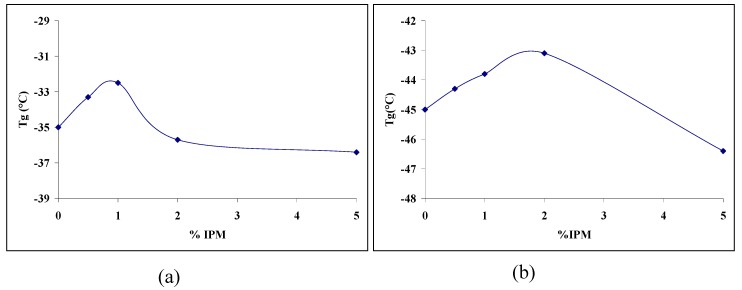
Glass transition temperature of (a) PEU-1 series (b) PEU-2 series (results of duplicate measurement by DMA, ±0.1 ºC).

DMA was used to characterise the compositional dependence of local, short range, motion, as well as any larger scale cooperative segmental motion that may exist in segmented polyurethanes. Miscibility of IPM and the extent of domain separation were determined from the glass transition temperature of the soft segments, identified through DMA analysis. Previous studies have shown that as the molecular weight of the soft segment increases, the T_g_ value of PTMO shifts towards lower temperatures and the maximum becames sharper, without plasticizer [34]; the T_g_ values obtained from DMA measurements are illustrated in Figure 5.

Further evidence for increases in packaging efficiency in the blend can be obtained from the shape of the tan δ peak with increasing IPM content as explained by Hill *et al.* [35]. The half width of the tan δ peak remained almost constant with increasing IPM content, but intensity decreases and the maximum shifts to a higher temperature (not shown). This behaviour is consistent with a low energy loss and increased elasticity in the blends. 

The DMA and DSC results point to a change in the free volume of the soft segment on the addition of IPM based on shifts in thermal properties. The initial increase in glass transition temperature on addition of IPM, and the subsequent decrease at higher concentrations, reflects an initial anti-plasticization and a subsequent plasticization effect on the PEU soft segment. Anti-plasticization is a phenomenon that occurs in many polymers on addition of low levels of plasticizer. Under these conditions, Young’s modulus and tensile strength are observed to increase initially to a maximum and then to decrease as normal plasticization sets in, an observation made for polyurethane and PDMS blends [21,35]. The most accepted explanation for this anti-plasticization is that a small amount of plasticizer, below a threshold level, provides enough additional free volume (or lubrication) to the system to permit limited chain mobility. This can result in a greater degree of polymer-polymer interaction or realignment with the development of small increases in localized molecular order and chain packing [36]. For blends, this means that at the low IPM concentrations of 1-2 wt%, the IPM promoted improved packing of the PTMO chains that were associated with it. This can also be linked to the observed relationship between Young’s modulus and tensile strength with increasing plasticizer content, *vide infra*.

### 3.3. Mechanical Properties

**Figure 6:**
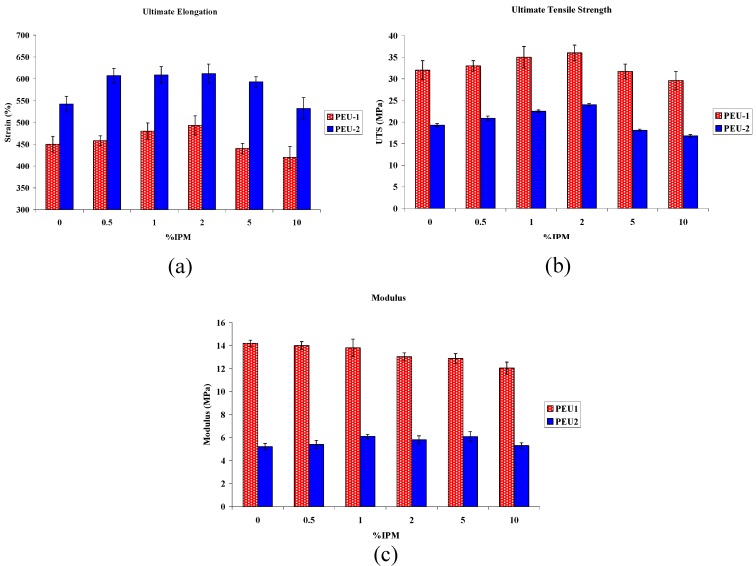
Effect of IPM content on mechanical properties of polyether urethanes (a) ultimate elongation; (b) ultimate tensile strength; (c) Young’s modulus.

Elastic modulus, ultimate tensile strength, and percent elongation at break were determined from stress-strain plots for each sample. All samples demonstrated elastomeric behaviour in the tests. Figure 6 shows the relationship between IPM content and mechanical properties of the films. A number of factors, including the specific chemistry and polymer molecular weight were expected to impact significantly on mechanical properties. Incorporation of IPM, most likely, resulted in a plasticized polymer. This is shown by an increase in elongation to break up to 2wt% IPM. The percent elongation at break exhibited an increase with 1 and 2 wt % IPM, indicating that softer materials were formed upon blending with IPM.

With respect to mechanical properties, it was found that both elongation at break and the tensile strength of the polyurethane increased slightly on addition of IPM up to 2 percent, but decreased when more IPM was added. The reduced mechanical properties at higher concentrations of IPM might be due to saturation of IPM in the PEU matrix with a separated IPM liquid phase which affected mechanical characteristics. 

Taken together, the trends in all of the above properties as a function of IPM content, are as follows: (i) both polyurethane series show optimum plasticization at an IPM blend of 1-2 wt% (ii) the effect diminishes signifcantly at IPM concentration above the optimal (iii) the softer PEU-2 series shows a greater percentage improvement in mechanical properties (especially elongation) than the PEU-1 series compared to initial PEU.

It is proposed that IPM facilitates improved packing efficiency (similar to anti-plasticization) in the polyurethane soft domain, leading to improved material performance. Beyond the optimum 1-2 wt% IPM, phase separation becomes significant, plasticization sets in, and mechanical properties then diminish. A plasticizer also reduces the crystallinity of the PEU soft domain, and this in combination with the additional lubrication effect, could also account for any increase in ultimate elongation; such an effect has been observed for polyurethane blended with PDMS [35].

### 3.4. Contact Angle

**Figure 7:**
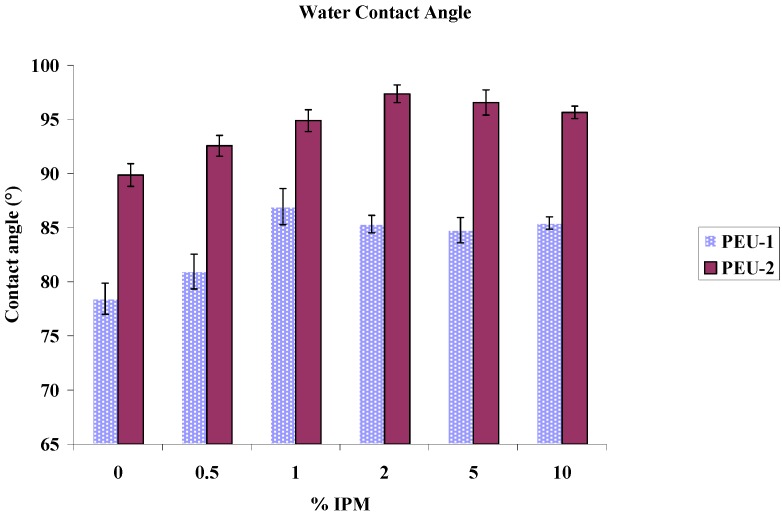
Water contact angle of IPM modified polyurethanes.

Water contact angles at blend film surfaces are shown in Figure 7. Contact angle on the surface of IPM-PEU-1 blend films increased from 78° to 87° with IPM content from 0 to 10 wt% to a maximum of ~87° at 1 wt%. A similar trend was observed for the PEU-2 series with contact angle increasing from 89° to 97° and a maximum of 97° at 2 wt% IPM. In both series, the contact angle has increased by adding IPM and has then levelled off. Greater soft segment content produced higher contact angles for the PEU-2 series and contact angle increased slightly by adding IPM in both series. This could be due to the hydrophobicity of an IPM rich phaseat the air-polymer interface.

### 3.5. Water Uptake

**Figure 8:**
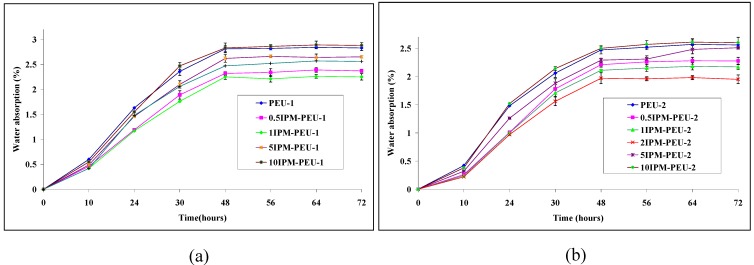
Water absorption of (a) PEU-1 series, (b) PEU-2 series after 72 hours soaking in deionised water at 37 °C.

Figure 8 displays the water absorption of the IPM-PEU systems. Equilibrium water uptake was achieved at about 36 hours for all samples. Water uptake of PEU samples decreased with increasing amounts of IPM added up to 2 wt%; this is presumably due to the inherent hydrophilicity of PTMO segments compared with those containing IPM. Furthermore, the PEU-2 samples, with higher PTMO content, exhibited less water absorption; the greater concentration of these less polar soft segments on the surface may have served as a diffusion barrier. For the PEU-2 series, maximum effect was seen at 2% IPM, so in both series the water uptake again coincides with the maximum effect on most of the other parameters.

The plasticized material presents a hydrophobic phase; a reversion of this trend would suggest that with IPM forming an independent second phase, its loss from PEU occurred allowing replacement by water. However, the higher chain packing obtained by loading 1-2 wt% IPM in the PEU-1 and PEU-2 series with the hydrophobicity of IPM could lead to reduced water uptake. The PEU-2 series with longer PTMO chains showed less water uptake compared with the PEU-1 series which shows the dominant role of the soft segment in water the absorption of polyurethane. PEU-1 samples with 1wt% IPM showed minimum water absorption as well as the 2 wt% IPM in the PEU-2 series. As it discussed above, this might be because of higher chain packing in these samples by addition of IPM up to optimum concentrations. The coincidence of maximum water contact angle with the lowest water uptake supportsthe above possibility. Results show that adding more than 5 wt% IPM in either PEU series did not reduce the water absorption. As was shown, IPM exists as a separate phase in polyurethane when its concentration is high, consequently it may leach out from the polymer and be substituted with water which will increase measured water absorption. The membrane stability of IPM and the biocompatibility of the blend will be investigated in next setof studies. 

## 4. Conclusions

In this study isopropyl myristate was used as an additive in polyether-urethane to enhance its properties for coating implantable micro electronics. Spectroscopic, thermal and mechanical properties as well as surface hydrophilicity and water absorption indicators confirmed that some benefit is derived using IPM, and this may be important over the longer term. The maximum for this effect is the wt 1-2% IPM. The mechanism is likely to be a change in free volume of the soft segments, their hydrophobic nature and the establishment of a single phase system below the wt 1-2% threshold for a separate IPM phase. The stability of this system and its tissue biocompatibility requires to be established in future work.

## References

[1] Hodgins D., Wasikiewicz J.M., Grahn M.F., Paul D., Roohpour N., Vadgama P., Silmon A. M., Cousins B., Verdon B. (2007). Biocompatible materials development for new medical implants. Med.l Device Technol..

[2] Hierold C., Clasbrummel B., Behrend D., Scheiter T., Steger M., Oppermann K., Kapels H., Landgraf E., Wenzel D., Etzrodt D. (1999). Low power integrated pressure sensor system for medical applications. Sensors Actuator. A-Phys..

[3] Receveur R.A.M., Lindemans F.W., de Rooij N.F. (2007). Microsystem technologies for implantable applications. J. Micromechanic. Microengineer..

[4] Dario P., Carrozza M.C., Benvenuto A., Menciassi A. (2000). Micro-systems in biomedical applications. J. Micromechanic. Microengineer..

[5] Donaldson P.E.K. (1989). Encapsulating microelectronic implants in one-part silicone rubbers. Med. Biol. Eng. Comput..

[6] Hodgins D., Bertsch A., Post N., Frischholz M., Volckaerts B., Spensley J., Wasikiewicz J.M., Higgins H., von Stetten F., Kenney L. (2008). Healthy aims: Development new medical implants and diagnostic equipment. IEEE Pervasive Comput..

[7] Wasikiewicz J.M., Roohpour N., Paul D., Grahn M., Ateh D., Rehman I., Vadgama P. (2008). Polymeric barrier membranes for device packaging, diffusive control and biocompatibility. Appl. Surf. Sci..

[8] Lamba N.M.K., Woodhouse K.A., Cooper S. (1997). Polyurethane in Biomedical Application.

[9] Wright J.I. (2006). Using Polyurethanes in Medical Applications. Med. Device Diagn. Ind..

[10] Morimoto N., Iwasaki Y., Nakabayashi N., Ishihara K. (2002). Physical properties and blood compatibility of surface-modified segmented polyurethane by semi-interpenetrating polymer networks with a phospholipid polymer. Biomaterials.

[11] Xu R.J., Manias E., Snyder A.J., Runt J. (2003). Low penneability biomedical polyurethane nanocomposites. J. Biomed. Mater. Res. A.

[12] Dube N., Al-Malaika S., Laroche G., Vermette P., Vermette P., Griesser H.J., Laroche G., Guidoin R. (2001). Additives in biomedical polyurethanes. Biomedical Applications of Polyurethanes.

[13] Krishna O.D., Kim K., Byun Y. (2005). Covalently grafted phospholipid monolayer on silicone catheter surface for reduction in platelet adhesion. Biomaterials.

[14] Wang X.D., Luo X., Wang X.F. (2005). Study on blends of thermoplastic polyurethane and aliphatic polyester: morphology, rheology, and properties as moisture vapor permeable films. Polym. Test..

[15] Morimoto N., Watanabe A., Iwasaki Y., Akiyoshi K., Ishihara K. (2004). Nano-scale surface modification of a segmented polyurethane with a phospholipid polymer. Biomaterials.

[16] Yoo H.J., Kim H.D. (2005). Characteristics of crosslinked blends of Pellethene((R)) and multiblock polyurethanes containing phospholipid. Biomaterials.

[17] Reddy S.M., Vadgama P.M. (1997). Surfactant-modified poly(vinyl chloride) membranes as biocompatible interfaces for amperometric enzyme electrodes. Anal. Chim. Acta.

[18] Pauliukaite R., Schoenleber M., Vadgama P., Brett C.M.A. (2008). Development of electrochemical biosensors based on sol-gel enzyme encapsulation and protective polymer membranes. Anal. Bioanal. Chem..

[19] Abraham M.H., Acree W.E. (2005). Characterisation of the water-isopropyl myristate system. I. J. Pharm..

[20] Gupta S., Moulik S.P. (2008). Biocompatible microemulsions and their prospective uses in drug delivery. J. Pharm. Sci..

[21] Bremner T., Hill D.J.T., Killeen M.I., Odonnell J.H., Pomery P.J., StJohn D., Whittaker A.K. (1997). Development of wear-resistant thermoplastic polyurethanes by blending with poly(dimethyl siloxane) .2. A packing model. J. Appl. Polym. Sci..

[22] Stetzler R.S., Smullin C.F. (1962). Determination of hydroxyl number of polyoxyalkylene ethers by acid-catalyzed acetylation. Anal. Chem..

[23] McCarthy S.J., Meijs G.F., Mitchell N., Gunatillake P.A., Heath G., Brandwood A., Schindhelm K. (1997). *In-vivo* degradation of polyurethanes: transmission-FTIR microscopic characterization of polyurethanes sectioned by cryomicrotomy. Biomaterials.

[24] Bai C.Y., Zhang X.Y., Dai J.B., Zhang C.Y. (2007). Water resistance of the membranes for UV curable waterborne polyurethane dispersions. Prog. Org. Coating..

[25] Garton A. (1992). Inferared Spectroscopy of Polymer Blends, Composites and Surfcaes.

[26] Movasaghi Z., Rehman S., Rehman I.U. (2008). Fourier transform infrared (FTIR) spectroscopy of biological tissues. Appl. Spectrosc. Rev..

[27] Movasaghi Z., Rehman S., Rehman I.U. (2007). Raman spectroscopy of biological tissues. Appl. Spectrosc. Rev..

[28] Teo L.S., Chen C.Y., Kuo J.F. (1997). Fourier transform infrared spectroscopy study on effects of temperature on hydrogen bonding in amine-containing polyurethanes and poly(urethane-urea)s. Macromolecules.

[29] Mattia J., Painter P. (2007). A comparison of hydrogen bonding and order in a polyurethane and poly(urethane-urea) and their blends with poly(ethylene glycol). Macromolecules.

[30] Koberstein J.T., Galambos A.F. (1992). Multiple melting in segmented polyurethane block copolymers. Macromolecules.

[31] Ambrose R.J. (1990). Thermoplastic Polymer Additives - Theory and Practice.

[32] Eddy S., Warriner K., Christie I., Ashworth D., Purkiss C., Vadgama P. (1995). The modification of enzyme electrode properties with nonconducting electropolymerized films. Biosens. Bioelectron..

[33] Vadgama P., Ahmed S. Modified polyurethane membrane sensors and analytical methods. US Patent.

[34] Lin Y.-H., Chou N.-K., Chen K.-F., Ho G.-H., Chang C.-H., Wang S.-S., Chu S.-H., Hsieh K.-H. (2007). Effect of soft segment length on properties of hydrophilic/hydrophobic polyurethanes. Polym. Int..

[35] Hill D.J.T., Killeen M.I., Odonnell J.H., Pomery P.J., StJohn D., Whittaker A.K. (1996). Development of wear-resistant thermoplastic polyurethanes by blending with poly(dimethyl siloxane). 1. Physical properties. J. Appl. Polym. Sci..

[36] Mascia L. (1989). Thermoplastics:Materials Engineering.

